# Synthetic and Nanotechnological Approaches for a Diagnostic Use of Manganese

**DOI:** 10.3390/molecules27103124

**Published:** 2022-05-13

**Authors:** Maddalena Sguizzato, Petra Martini, Lorenza Marvelli, Walter Pula, Markus Drechsler, Martina Capozza, Enzo Terreno, Lucia Del Bianco, Federico Spizzo, Rita Cortesi, Alessandra Boschi

**Affiliations:** 1 Department of Chemical, Pharmaceutical and Agricultural Sciences (DoCPAS), University of Ferrara, 44121 Ferrara, Italy; sgzmdl@unife.it (M.S.); walter.pula@edu.unife.it (W.P.); alessandra.boschi@unife.it (A.B.); 2 Biotechnology Interuniversity Consortium, Ferrara Section, University of Ferrara, 44121 Ferrara, Italy; 3 Department of Environmental and Prevention Sciences, University of Ferrara, 44121 Ferrara, Italy; petra.martini@unife.it; 4 INFN—Laboratori Nazionali Legnaro, National Institute of Nuclear Physics, Viale dell’Università, 2, 35020 Legnaro, Italy; 5 Key Lab “Electron and Optical Microscopy”, Bavarian Polymer Institute (BPI), University of Bayreuth, 95440 Bayreuth, Germany; markus.drechsler@uni-bayreuth.de; 6 Molecular & Preclinical Imaging Center, Department of Molecular Biotechnologies and Health Sciences, University of Torino, 10126 Torino, Italy; martina.capozza@unito.it (M.C.); enzo.terreno@unito.it (E.T.); 7 Department of Physics and Earth Science, University of Ferrara, 44122 Ferrara, Italy; lucia.delbianco@unife.it (L.D.B.); federico.spizzo@unife.it (F.S.)

**Keywords:** lipid-based nanosystems, liposomes, manganese, PET/MRI, magnetic susceptibility

## Abstract

The development of multimodal imaging techniques such as positron emission tomography (PET) and magnetic resonance imaging (MRI) allows the contemporary obtaining of metabolic and morphological information. To fully exploit the complementarity of the two imaging modalities, the design of probes displaying radioactive and magnetic properties at the same time could be very beneficial. In this regard, transition metals offer appealing options, with manganese representing an ideal candidate. As nanosized imaging probes have demonstrated great value for designing advanced diagnostic/theranostic procedures, this work focuses on the potential of liposomal formulations loaded with a new synthesized paramagnetic Mn(II) chelates. Negatively charged liposomes were produced by thin-layer hydration method and extrusion. The obtained formulations were characterized in terms of size, surface charge, efficiency of encapsulation, stability over time, relaxivity, effective magnetic moment, and in vitro antiproliferative effect on human cells by means of the MTT assay. The negatively charged paramagnetic liposomes were monodisperse, with an average hydrodynamic diameter not exceeding 200 nm, and they displayed good stability and no cytotoxicity. As determined by optical emission spectroscopy, manganese complexes are loaded almost completely on liposomes maintaining their paramagnetic properties.

## 1. Introduction

Manganese (Mn) is an essential bio-element for humans, is a cofactor of many enzymes, and is involved in multiple and fundamental cellular activities, including macronutrient metabolism, bone formation, and free-radical defense systems [1]. Notwithstanding the need of manganese for various physiological activities, its excess in the human body can be toxic [2,3].

Manganese is a *d*-transition metal belonging to the seventh group of the periodic ta-ble; in its +2 and +3 oxidation states, it shows paramagnetic properties, making it a potential candidate for MRI applications.

MRI relaxation times are directly proportional to the concentration of Mn^2+^ ions in the tissues. A higher amount of Mn^2+^ results in a stronger and more detectable contrast, leading to better image quality [4]. Currently, the main limiting factor for the use of Mn^2+^ ion as a contrast agent in clinical practice is its neurotoxic effects [4,5]. Therefore, the critical point in employing Mn as a contrast agent is the need to minimize side-effects by using the lowest dose sufficient to be detected by MRI.

To limit the toxicity, the manganese ion may be masked before administration, for example, by using chelating agents.

The Mn–DPDP complex (mangafodipir, Teslascan^TM^) is the only manganese-based contrast agent clinically approved to study liver lesions through absorption by hepatocytes [6].

The complex is formed by the coordination of the di-pyridoxal diphosphate ligand to the Mn^2+^ ion, and, once injected, it is taken up by healthy hepatocytes where it experiences a transmetallation by intracellular proteins, resulting in a significant enhancement of T_1_ contrast. However, in 2012 mangafodipir was withdrawn from European trade for commercial and toxicity reasons [7].

In recent years, the research for developing new Mn^2+^ complexes as MRI agents has received a strong input after concerns about the safety of Gd(III) complexes [8,9].

This led to many literature reports aimed at developing small-sized Mn(II) complexes as potential contrast agents as a valuable alternative to Gd(III) [10].

In the framework of the METRICS project (an acronym for multimodal PET/MRI imaging with cyclotron-produced ^51/52^Mn isotopes), which aims to develop the technology needed to obtain a cyclotron-driven production of ^52/51^Mn PET radionuclides and to achieve PET/MRI hybrid imaging by taking advantage of both paramagnetic and radio-active properties shown by manganese isotopes, we started an investigation into the class of dithiocarbamate ligands with the purpose of developing a new class of mononuclear manganese(II)-based agents for medical applications. These preliminary studies showed that Mn(II)(S_2_CNR_2_)_2_ complexes (R = Et or CH_2_CH_2_OEt) are poorly soluble in physiological conditions, and, if weathered or not properly stored in a controlled atmosphere, they convert into the tris-substituted complexes Mn(II)(S_2_CNR_2_)_3_ over time [11].

A potential strategy to exploit manganese bimodal contrast properties by limiting the drawback of a poor water solubility is represented by their loading into liposomes, known for their wide range of applications in the biomedical field, including in diagnostics and theranostics [12,13,14,15]. Accordingly, we recently reported a pre-formulation study of anionic liposomes as delivery systems for manganese [16].

In order to overcome potential toxicity, solubility, and stability problems, in the present study, the production and characterization of anionic liposomes as delivery systems for a new class of manganese complexes were investigated.

**Figure 1:**
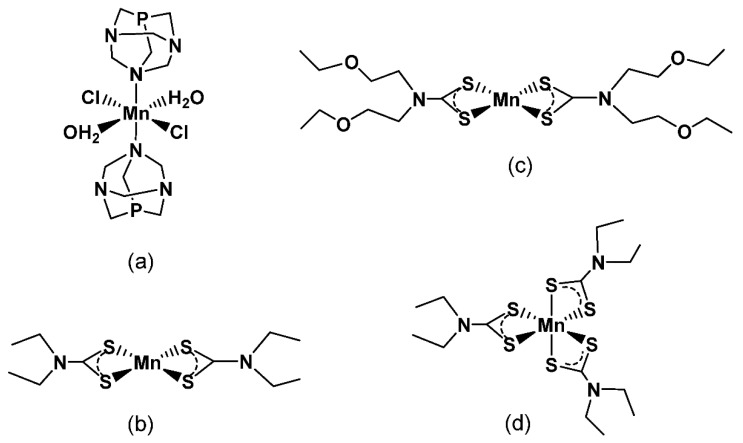
Chemical structure of Mn complexes: MP (**a**), MDE2 (**b**), MDB (**c**), and MDE3 (**d**).

In particular, the following Mn(II)/Mn(III) complexes characterized by different water solubility were considered: [Mn(II)(PTA)(Cl)_2_(H_2_O)_2_] (MP) (PTA = 1,3,5-triaza-7-phosphaadamantane), Mn(II)(S_2_CNR_2_)_2_ (R = Et, MDE2; CH_2_CH_2_OEt, MDB), and Mn(III)(S2CNEt2)_3_ (MDE3) (Figure 1). Notably, the presence of the adamantan phosphine PTA in MP allowed obtaining a more water-soluble complex. On the other hand, the use of dithiocarbamate ligands made it possible to compare the behavior of more lipophilic compounds.

## 2. Results and Discussion

### 2.1. Preparation of the Mn Derivatives

The manganese complexes were prepared and characterized according to the procedure reported in the literature [17]. Briefly, all complexes were prepared by reacting the Mn(II) salt with the PTA or dithiocarbamate ligands.

The synthesis of MDE2 and MDB was performed under inert conditions by reacting the Mn(II)Cl_2_·4H_2_O complex with two equivalents of the sodium salts of the dithiocarbamate ligand (Na[(CH_3_CH_2_)_2_-N-C(=S)S]·3H_2_O or Na[(CH_3_CH_2_OCH_2_CH_2_)_2_-N-C(=S)S] ·3H_2_O), after careful purging of all the reaction containers and water solvent by nitrogen bubbling and using the Schlenk technique. The preparation of the MDE3 complex was performed under open atmosphere by reaction of MnCl_2_ with three equivalents of Na[(CH_3_CH_2_)_2_-N-C(=S)S]·3H_2_O, in water solution.

All complexes were characterized with elemental analysis, ESI(+)-MS, UV/Vis, and infrared (IR) analysis as previously reported [11]. In Figure 1, the chemical structures of the selected Mn compounds are reported.

### 2.2. Magnetic Characterization of the Produced Mn Derivatives

**Figure 2:**
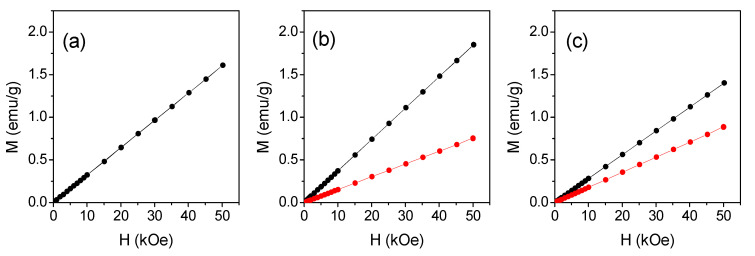
Magnetization (M) vs. magnetic field (H) plots measured at T = 300 K on MP (**a**), MDE2 (**b**), and MDB (**c**) powders as samples freshly prepared (black line) or aged 13 (**b**) and 3 (**c**) days (red line).

MP, MDE2, and MDB were analyzed as powders, and curves of magnetization (M) vs. magnetic field (H) were measured at T = 300 K (Figure 2).

The results obtained for the freshly prepared derivatives (black lines) clearly indicated a paramagnetic behavior. The slope of the curves represents the mass susceptibility, defined as χ = M/H (expressed in emu/g Oe), which is positive and independent from H in paramagnetic materials. Further analyses were repeated over time on the same samples stored in the open air, until the magnetic response no longer changed significantly. The magnetic behavior of MP did not change over time (Figure 2a). On the other hand, in the case of MDE2 and MDB derivatives, a progressive reduction in χ occurred over time as indicated by the measures performed after 13 and 3 days from preparation, respectively (red line, Figure 2b,c).

**Figure 3:**
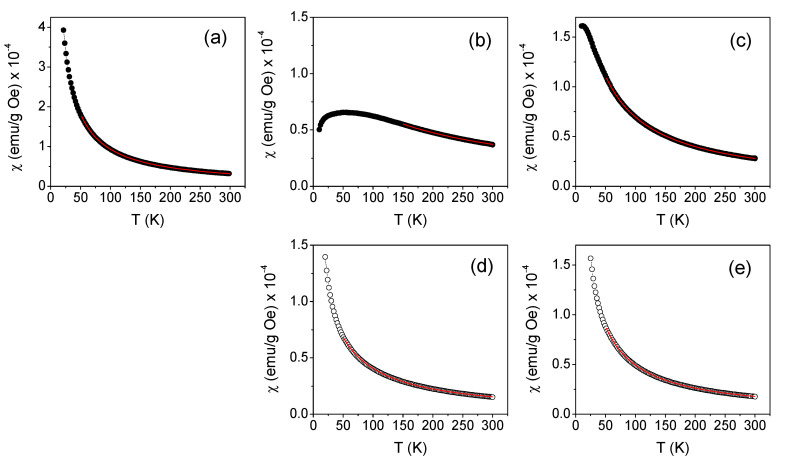
Curves of mass susceptibility (χ) as a function of temperature (T) measured on the freshly (**a**–**c**) or aged (**d**,**e**) prepared MP (**a**), MDE2 (**b**,**d**), and MDB (**c**,**e**) compounds, in H_appl_ = 10 kOe. The red lines are the fitting curves to the Curie–Weiss law.

The magnetization M was measured as a function of temperature between 10 K and 300 K in an applied magnetic field H_appl_ = 10 kOe. The results obtained for the three freshly prepared manganese derivatives are reported in Figure 3 (panels a–c), where the thermal evolution of χ is actually shown.

At T = 300 K, the values of χ were obviously equal to those derived from the M vs. H measurements (see Table 1).

The thermal dependence of the susceptibility is expected to follow the Curie-Weiss law for paramagnetic materials, as expressed in Equation (1) [18].

(1) $$\chi =\frac{0.125}{\mathrm{MW}}\;\;\;\frac{\mu^{2}{}_{\mathrm{eff}}}{T\;–\;\theta }$$

where MW is the molecular weight, μ_eff_ is the effective magnetic moment per molecule of paramagnetic material (expressed in Bohr magneton units, BM), and θ (which has the dimension of a temperature) gives information on the type and strength of magnetic interactions between the magnetic moments in the paramagnetic regime.

Since each investigated compound contains a paramagnetic Mn ion, then μ_eff_ coincides with the magnetic moment of the Mn ion and, according to Equation (1), is

μ_eff_ = 2.828 × [χ·MW·(T − θ)]^1/2^ (2)

In order to estimate μ_eff_ from the above relation, the value of θ is obtained by fitting the χ vs. T curves in Figure 3 to the Curie–Weiss law.

In the case of MP, χ followed the dependence 1/(T − θ) foreseen by the Curie–Weiss law in the whole investigated temperature range, unlike that observed for MDB and MDE2. In MDB, the susceptibility tended to a plateau value at a very low temperature. This behavior indicates the existence of magnetic interactions between molecular moments so strong as to determine the onset of a magnetic state that could no longer be described as purely paramagnetic. The same effect was even more marked in the case of MDE2, where the χ vs. T curve deviated substantially from the 1/T trend below ~150 K and exhibited a strong decrease below ~60 K. Therefore, the fitting of the χ vs. T curve to the Curie–Weiss law was performed for the whole investigated temperature range in the case of MP, whereas the 150–300 K and the 50–300 K intervals were considered for MDE2 and MDB, respectively (the fitting curves are the continuous red lines in Figure 3).

Table 2 reports the values of θ at 300 K, χ, and μ_eff_ for the three freshly prepared compounds, as well as for MnCl_2_, used as a reference compound. The χ vs. T curve measured on this sample is shown in Figure S1. The diamagnetic contribution to χ provided by the elements in the compounds other than Mn was smaller than the indicated experimental error.

The μ_eff_ values were obtained from Equation (2) using the predicted MWs. For MnCl_2_, μ_eff_ = (5.9 ± 0.2) BM is in excellent agreement with the value expected for the Mn(II) ion in the high-spin state (i.e., 5.92 BM). The values measured in MP and MDE2 were also consistent with the high-spin state of Mn(II), within experimental error, while a larger value was measured in MDB.

The parameter θ was negative for the three compounds, indicating that the magnetic interactions between the magnetic moments were antiferromagnetic. The absolute value of θ is a measure of the magnitude of the magnetic interactions; therefore, it turns out that they were weak in MP and definitely strong in MDE2.

Measurements of χ vs. T were also performed on the aged MDE2 and MDB samples; which, based on the analysis over time of the M vs. H curves (Figure 2b,c), were stable. The curves obtained for MDE2 and MDB are shown in Figure 3d,e, respectively. Unlike that observed in the freshly prepared samples, their trend appeared more compatible with the Curie–Weiss law across the spanned temperature range. The values of χ at T = 300 K, θ, and μ_eff_, obtained by the analysis of these curves are reported in Table 2. A strong decrease in the three parameters was experienced. The value of μ_eff_ for MDB was consistent with that for the high-spin state of Mn^3+^ (i.e., 4.89 BM). As for MDE2, a μ_eff_ as low as ~3.4 BM was estimated. This is in agreement with the previous observations that Mn(II) compounds are oxidized over time to Mn(III) if not stored under controlled conditions in the absence of oxygen [11].

### 2.3. Liposome Preparation and Characterization

In our previous study, a pre-formulatory study allowed us to select NLS and SLL as optimal anionic surfactants for the delivery of both hydrophilic and lipophilic manganese-based compounds [16].

In the present study, anionic liposomes with NLS and SLL, namely, LN and LS, were produced as delivery systems for the Mn complexes here proposed. Table 2 reports the composition of the prepared liposomal formulations.

The loading of manganese complexes to liposomes occurred in relation to the lipophilic or hydrophilic nature. In the case of complexes MDE2, MDE3, and MDB, they were added to the organic phase before the film formation, while, in the case of MP, the compound was solubilized in the aqueous phase before the hydration step.

The produced formulations, summarized in Table 1, after a visual inspection, were characterized in terms of size and polydispersity. From a macroscopic point of view, they appeared as milky uniform suspensions. With the aim of decreasing vesicle size and increasing the homogeneity of the dispersed vesicles, the formulations were subjected to extrusion through a polycarbonate filter.

**Figure 4:**
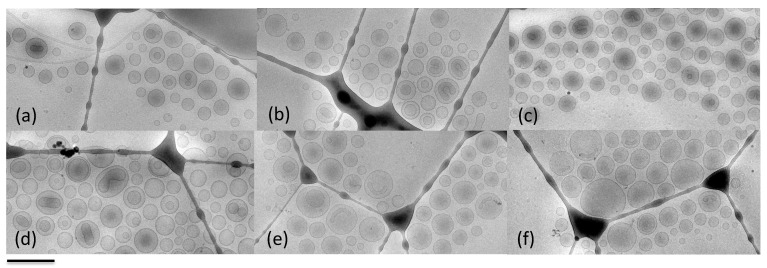
Cryo-TEM images of the produced liposomes visualized after extrusion: LS-MP (**a**), LS-MDE2 (**b**), LS-MDB (**c**), LN-MP (**d**), LN-MDE2 (**e**), and LN-MDB (**f**). Bar corresponds to 500 nm.

The successful formulation of the anionic liposomes was demonstrated with morphological analysis by Cryo-TEM (see Figure 4).

It can be noticed that, as expected, the formulations were mainly composed of quite homogeneous sized unilamellar vesicles, and the presence of either hydrophilic or lipophilic derivative did not affect the morphological structure of the liposomes. In addition, the use of the anionic surfactants led to the physicochemical stability of vesicles thanks to the negative charges on the surface, while interacting with Mn^2+^ and Mn^3+^ ions.

**Figure 5:**
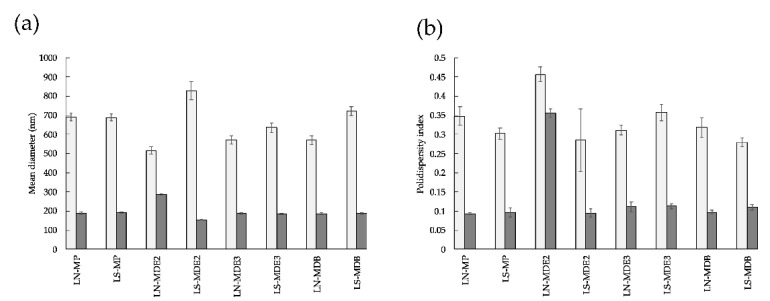
Dimensional parameters of the produced anionic liposomes obtained before (light gray) and after extrusion (dark gray). Mean diameters are expressed as Z-average (**a**) and dispersity index values (**b**).

The data concerning vesicle size and dispersion before and after extrusion are summarized in Figure 5.

As reported in Figure 5a, after production, liposomes with great mean diameters, between 515 and 820 nm, were obtained as a function of the surfactant used and the type of encapsulated complex. However, after extrusion, all the formulations presented Z-averages around 190 nm, excluding LN-MDE2, confirming the efficacy of the process in decreasing vesicle size due to the influence of the filter pores.

The extrusion strategy also influenced the dimensional distribution of the vesicles. The obtained dispersity index values were around 0.1, indicating a monomodal distribution and a homogeneous population of small vesicles. The only exception was represented by LN-MDE2, showing a polydispersity value typical of a broad distribution of liposomal vesicles, corroborated by the high Z-average value. This behavior could be ascribed to the unsuccessful entrapment of this lipophilic compound within the phospholipid bilayer after extrusion. Indeed, at the end of the procedure, a brown precipitate was found on the polycarbonate membrane, suggesting the possible expulsion of the drug from liposomes during the process.

Moreover, taking into consideration the oxidative reaction on MDE2 occurring in contact with air and leading to structural modifications, the preparation of LN-MDE2 was realized directly after synthesis, in order to reduce the time of air exposure, exploiting the advantage of liposome in protecting the encapsulated drug from degradation [19].

#### Physical Stability of Loaded-Liposomes

The storage stability in terms of size and dimensional distribution of extruded liposomes containing manganese complexes was evaluated for 60 days by means of PCS. The Z-average and PdI values are reported in Table 3.

As reported, all the formulations maintained their sizes around 190 nm during the investigated period. Concerning the dimensional distribution, values were stable around 0.1, confirming the presence of a uniform population of vesicles not subjected to aggregation phenomena.

As mentioned above, despite the extrusion process, LN-MDE2 showed large dimensions around 300 nm and a dispersion index of 0.36. The high polydispersity value could be responsible for instability phenomena over time and, therefore, highly probable adverse effects following future administrations. On the other hand, the presence of the SLL surfactant gave rise to more stable and homogeneous loaded liposomes with a restricted size distribution, even in the case of MDE2; thus, the interaction of this manganese complex with NLS could be responsible for the dimensional variability.

### 2.4. Encapsulation Efficiency of Synthesized Mn Complexes in Anionic Liposomes

The encapsulation efficiency of the different complexes in LN and LS was evaluated by ICP-OES analysis, identifying Mn ions in the formulation. In order to verify the association of the different compounds with the lipid bilayer of vesicles, ultracentrifugation was used to separate the lipid phase from aqueous phase, and the amount of Mn ions in the lipid phase was quantified. Indeed, as recently discussed, manganese was successfully encapsulated into anionic liposomes and retained by its lipid portion possibly intercalated or associated to the phospholipid bilayer, depending on the hydrophilic or lipophilic characteristics of the complex [16]. Indeed, FT-IR spectroscopy analysis revealed that hydrophilic complexes could establish electrostatic interactions between Mn^2+^ and the negatively charged phospholipid surface.

**Figure 6:**
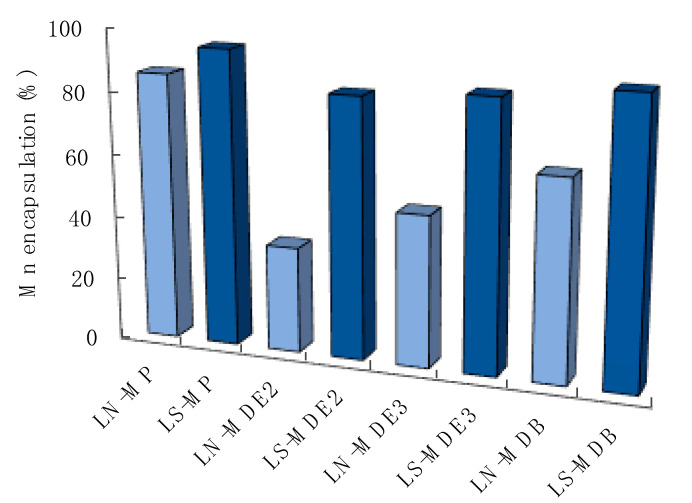
Manganese complexes encapsulated in the lipid phase of LN (light blue) and LS (blue) after ultracentrifugation, expressed as a percentage with respect to total amount of manganese used in the formulation.

Figure 6 displays the amount of Mn retained by the lipid phase of LN and LS, expressed as a percentage with respect to the total amount of complex used in the formulation. Overall, LS demonstrated a higher retention of manganese than LN, showing greater EE values, i.e., 94%, 82%, 85%, and 89% for MP, MDE2, MDE3, and MDB, respectively. The formulation did not exhibit significant differences in loading hydrophilic or lipophilic manganese compounds, resulting in a suitable system to convey all the synthesized complexes. It can be supposed that the hydrophilic complexes were able to establish electrostatic interactions between the Mn^2+^ ion and the phospholipid surface, allowing the retention of the complexes. To this aim, the ζ potential of unloaded and MP-loaded LS and LN was measured, with MP being the more hydrophilic manganese complex here considered. Notwithstanding the maintenance of a net negative charge of the systems, the obtained results confirmed previous published data [16] and indicated a reduction in ζ potential after MP loading, from −67.12 ± 0.64 mV to −51.18 ± 1.83 mV for LS and from −57.77 ± 0.26 mV to −49.21 ± 1.42 mV for LN, corroborating our hypothesis and the data obtained for liposomes loaded with manganese chloride [16].

The same performance did not occur for LN. Apart from MP, where 85% of manganese was retained by the formulation, for the other compounds, the EE was lower than 63%. In particular, in the case of MDE2, the lowest percentage corresponding to 33% corroborated the PCS result and the possible degradation or instability of the complex. These results suggested LS as the most effective formulation for the delivery of manganese complexes.

### 2.5. Magnetic and Relaxometric Investigations on Liposomes Loaded with Mn Compounds

**Figure 7:**
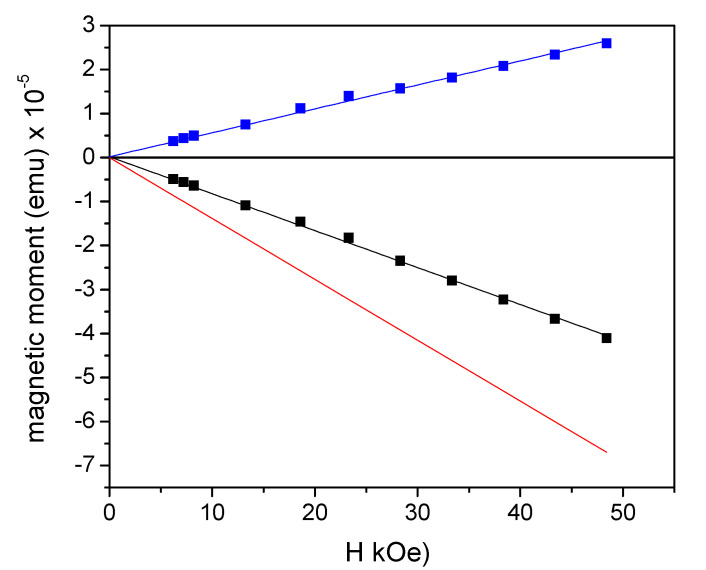
Magnetic moment measured as a function of H, at T = 300 K, on empty (red line) and MDB-loaded LS (black line). The blue line is the magnetic response of the sole MDB.

In order to obtain information about the magnetic properties of the prepared liposomes, some studies were conducted. Specifically, magnetic analysis was carried out to evaluate the actual amount of MDB loaded on liposomes with a nominal concentration of 0.28 mg/mL. For this preparation the aged MDB compound was used (Figure 2c). First, 100 μL of solution was dropped in a suitable sample holder for SQUID and left in open air until the water evaporated completely. Then, the magnetic moment of this dried LS-MDB sample was measured as a function of H, at T = 300 K. A dried sample of empty LS was also measured in order to disentangle the paramagnetic signal of MDB from the total one. According to the MDB magnetization at T = 300 K and H = 50 kOe (0.89 ± 0.03 emu/g, red line in Figure 2c), the expected magnetic moment of the sole MDB contained in the sample was (2.5 ± 0.2) × 10^−5^ emu, at H = 50 kOe. In the results reported in Figure 7, the black and red lines are the magnetic moment vs. H for the dried MDB-loaded and empty LS, respectively; the blue line is the magnetic response of the sole MDB, obtained by subtracting the diamagnetic contribution of the empty LS from the total signal. The magnetic moment at H = 50 kOe for the sole MDB was (2.6 ± 0.3) × 10^−5^ emu, in line with the expectation. Therefore, the MDB concentration, as assessed by the magnetic analysis, was consistent with the nominal one. The whole measurement procedure was repeated on three dried LS-MDB samples. The value of MDB magnetic moment was the same in the three cases, within experimental error, indicating a homogeneous presence of the compound in the liposome solution. The magnetic response of the three samples was monitored over time, and no changes were recorded.

**Figure 8:**
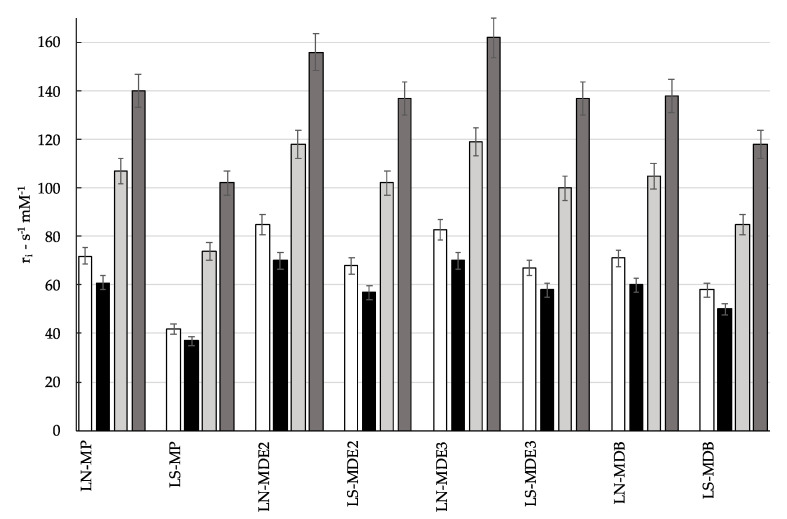
Longitudinal (r_1_; white, black) and transverse (r_2_; light gray, dark gray) relaxivity values of the indicated liposomes formulation measured at 25 °C in two magnetic fields: 0.47 T (Larmor frequency 20 MHz, white and light gray) and 1.4 T (Larmor frequency 60 MHz, black and dark gray).

The produced liposomal samples were subjected to a basic relaxometric investigation. Figure 8 shows the longitudinal (r_1_; white, black) and transverse (r_2_; light gray, dark gray) relaxivity values normalized to a 1 mM concentration of Mn(II). Measurements were conducted at 25 °C in two magnetic fields: 0.47 T (white and light gray) and 1.4 T (black and dark gray).

Overall, the relaxivity values were quite high, in line with paramagnetic centers experiencing slow-tumbling motions, thus clearly confirming the interaction between the metal center and the liposomal bilayer. For all samples, the longitudinal relaxivity values ranged from 40 to 85 s^−1^·mM^−1^, showing values at 1.5 T lower than 0.5 T, as predicted by the theory of the paramagnetic relaxation for systems with a restricted rotational motion. On the other hand, transverse relaxivities, as expected, were higher than the corresponding r_1_ values, with a direct correlation with the magnetic field. Few reports have been published so far on liposomes loaded with Mn^2+^ as free ion or complexed. In the former case, the application was in the field of MRI-guided imaging of drug release, where it was observed that the interaction of Mn^2+^ ions with phospholipids of the liposome bilayer led to a significant r_1_ enhancement to ca. 40 s^−1^·mM^−1^ at 0.5 T, but 35 °C [20]. More recently, Mulas et al. reported the relaxivity values of liposomes incorporating amphiphilic Mn^2+^ polyaminopolycarboxylic (acyclic and macrocyclic) complexes with very high thermodynamic stability. In addition to highlighting the active role of the metal complexes to drive size and self-assembling behavior of the nanoparticles, r_1_ values lower than 20 s^−1^·mM^−1^ were observed at 0.5 T and 25 °C [15].

Therefore, the relaxometric characterization of the studied liposomal formulations suggests that the Mn^2+^ center is not completely coordinated by its chelators; it is also involved in binding the (inner and/or outer) surface of the nanovesicular carriers.

### 2.6. Effect of Loaded Liposomes on Cultured Cells

The preliminary study conducted on plain LP and unloaded LN and LS demonstrated the safety of the formulations at different concentrations with a dose-dependent profile [16]. In this study, with the aim of verifying the effect of the encapsulated complexes in LN and LS aimed at screening of the formulations, HaCaT cells were selected as a human nontumor cell line. The cells were treated with the selected preparations and appropriately diluted; then, cell viability was evaluated with respect to untreated cells considered as 100% viable. Due to the dimensional instability and low EE, LN-MDE2 was not selected for this experiment.

**Figure 9:**
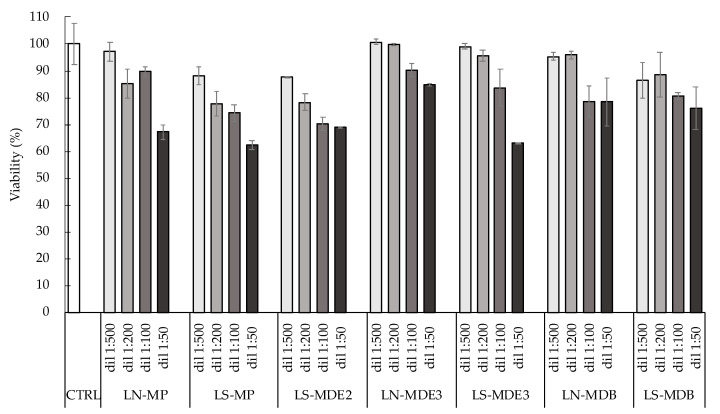
Effect of loaded LN and LS on cultured cells in vitro evaluated by MTT. Data are the means of three independent experiments ± SD conducted in triplicate.

As displayed in Figure 9, all the liposomal formulations showed a dose-dependent profile, in agreement with the results previously discussed. These data suggested that the encapsulation of manganese complexes did not affect the in vitro effect of LN and LS.

It should be noted that, in the case of both hydrophilic and lipophilic synthesized manganese compounds, the viability at the highest concentration tested was greater than 63%. This result was expected in the case of MP, while, in the case of MDE2, MDE3, and MDB, these data corroborate the relaxometric results, suggesting the association/partial intercalation of the encapsulated molecules to the phospholipid bilayer/surface, guaranteeing their protection from degradation, as well as the protection of treated cells from toxicity.

The use of charged surfactants in liposomes represents a suitable strategy to increase the formulation stability, for instance, to stabilize vesicle dimensional distribution, to increase zeta potential and, consequently, decrease vesicle coalescence, and to establish a stronger interaction with the cell membrane or cell components [21,22,23,24].

This preliminary study focused on the evaluation of the suitability of both new manganese compounds and the way to deliver them, allowing us to select some formulations. However, future studies will be designed in order to test the toxicity effects after administration by means of in vivo experiments.

## 3. Materials and Methods

### 3.1. Materials

Mn(II)Cl_2_·4H_2_O and Na[(CH3CH2)2-N-C(=S)S]·3H2O were obtained from Aldrich Chemical (Darmstadt, Germany), Na[S2CN(CH2CH2OEt)2] was obtained from Alchemy (Baricella, Italy), and cholesterol (CH) was obtained from Merck-Aldrich (Milano, Italy). *N*-Lauroylsarcosin sodium salt (NLS) was from Fluka Chemie AG (Buchs, Switzerland). Sodium lauroyl lactylate (SLL) was from Zschimmer & Schwarz Italiana S.p.A. (Vercelli, Italy) and Stepan Europe S.A.S. (Voreppe, France). Lastly, soybean phosphatidylcholine Phospholipon 90G (PC) was from Lipoid AG (Steinhausen, Switzerland). Solvents of analytical grade were from Merck Serono S.p.A. (Roma, Italy). All other materials and solvents of high-purity grade were from Sigma-Aldrich (St Louis, MO, USA). The heterocyclic phosphine 1,3,5-triaza-7-phosphaadamantane (PTA) and the Mn complexes MP, MDE2, MDE3, and MDB were prepared according to the procedure reported in the literature [11,17].

### 3.2. Liposomes Preparation and Characterization

Anionic liposomes with NLS and SLL, namely, LN and LS, were prepared by direct hydration followed by extrusion. Precisely, an organic solution of methylene chloride and methanol (1:1 *v*/*v*) containing PC, CH, and NLS or SLL in a 4:2:1 molar ratio to give a final concentration of 25 mg/mL was prepared. Afterward, the organic mixture was evaporated under vacuum (70 bar, rotation speed 3 for 40–45 min) by means of a Rotavapor R-200 (Buchi Italia, Cornaredo, Italy), and the obtained film was hydrated with 5 mL of water, swirled, and sonicated for 5 min. The addition in the selected formulation of manganese-based compounds (reaching a final theoretical content of 500 μM) was carried out on the basis of their chemical characteristics either in organic phase (lipophilic) or in water during the hydration step (hydrophilic). 

Liposomes were then subjected to extrusion leading to vesicles with a homogeneous size distribution [25]. Precisely, each liposome dispersion was extruded fivefold through two stacked polycarbonate filters with 0.2 µm pore size (Nucleopore Corp, Pleasanton, CA, USA.), supported by polyester drain disc using an Extruder (Lipex Biomembranes, Vancouver, Canada) and 10–20 bar of nitrogen pressure [26]. After the process, liposomes were collected and stored for further studies.

Liposome size was measured by mean of Zetasizer Nano S90 (Malvern Instr., Malvern, UK) equipped with a 5 mW helium neon laser with a wavelength output of 633 nm on aqueous diluted liposome samples (1:20 by volume). Plasticware was cleaned with detergent washing and rinsed twice with milliQ water. Measurements were made at 25 °C at an angle of 90°, with a run time of ~180 s. Data were interpreted using the “CONTIN” method [27].

The ζ potential measurements were carried out by means of a Zetasizer Ultra (Malvern Panalytical Ltd., Malvern, UK). All the samples were diluted in disposable capillary cells (DTS 1080, Malvern) with deionized water (1:20 *v*/*v*), and the analysis was conducted at 25 °C. Values were obtained from three independent experiments performed in triplicate.

Liposome morphology was investigated by means of cryo-transmission electron microscopy (cryo-TEM). Liposome samples were vitrified and transferred to a Zeiss EM922Omega transmission electron microscope for imaging using a cryoholder (CT3500, Gatan Inc., Pleasanton, CA, USA), as previously described [28,29]. Sample temperature was maintained below −175 °C throughout the visualization. Specimens were examined with doses of about 1000–2000 e/nm^2^ at 200 kV. Images were recorded digitally by a CCD camera (UltraScan 1000, Gatan Inc., Pleasanton, CA, USA) using GMS 1.4 software (Gatan Inc., Pleasanton, CA, USA) as an image processing system.

### 3.3. Optical Emission Spectrometry (ICP-OES)

Optical emission spectrometry (ICP-OES) is an analytical technique used for quantitative and qualitative determination of metal ions in solution. Therefore, each sample was subjected to the ICP-OES to determine the MN concentration actually present. The encapsulation efficiency (EE) of manganese in LN and LS was determined by ultracentrifugation; specifically, 500 μL samples were loaded in a centrifugal filter (Microcon centrifugal filter unit YM-10 membrane, NMWCO 10 kDa, Sigma-Aldrich, St. Louis, MO, USA) and subjected to ultracentrifugation (SpectrafugeTM 24D Digital Microcentrifuge, Woodbridge, NJ, USA) at 8000 rpm for 20 min. Then, the lipid phase was analyzed. All measurements were made with an ICP-OES device (Optima 3100 XL, Perkin-Elmer, Shelton, USA) equipped with an axial torch, segmented array charge-coupled device detector, and low-flow GemCone nebulizer with cyclonic spray chamber for sample introduction and choosing, among the several wavelengths, the readings at 259.372 nm. Each sample was prepared twice and subjected to analysis; the analyzed volume was 20 µL. For each condition, the average of the absorbance values obtained was calculated, and the manganese concentration was obtained by comparison with a calibration curve obtained after measuring known concentrations of the metal ion [30]. The EE was determined as follows:

EE = (Mn_LIPID_ (mM)/Mn_TOTAL_) × 100 (3)

where Mn_LIPID_ corresponds to the concentration of manganese in the lipid phase measured by ICP-OES, and Mn_TOTAL_ is the concentration of manganese used in the formulation.

### 3.4. Magnetic Measurements

The magnetic measurements were carried out using a superconducting quantum interference device (SQUID) magnetometer (Quantum Design GmbH, Darmstadt, Germany) operating with a maximum applied field H = 50 kOe. The instrument allows measuring the sample’s magnetic moment with 10^−7^ emu sensitivity. To calculate the specific magnetization (M = magnetic moment/sample mass, expressed in emu/g), the weight of the sample (few milligrams) was measured with a precision of 10^−5^ g.

### 3.5. Relaxometric Investigations

The longitudinal (R_1_ = 1/T_1_) and transverse (R_2_ = 1/T_2_) relaxation rates were measured at 25 °C at two magnetic field strengths, 0.47 T and 1.4 T (corresponding to 20 MHz and 60 MHz proton Larmor frequency), on a Stelar Spinmaster spectrometer working at adjustable field. T_1_ values were measured using the standard inversion recovery sequence (16 experiments, two scans) with a typical radio frequency 90° pulse length of 3.5 μs. T_2_ values were measured using a standard CPMG sequence (2048 sampled echoes, 16 scans, two averages). Temperature was controlled with a Stelar VTC-91 heater airflow equipped with a copper–constantan thermocouple (uncertainty of ±0.1 K). The relaxation rates of the solutions containing the paramagnetic complexes were subtracted from the corresponding diamagnetic contributions and then divided by the millimolar concentration of Mn ions to obtain the normalized millimolar relaxivities (r_1_ and r_2_, in mM^−1^·s^−1^).

### 3.6. Cell Culture and Viability Test

Cell viability was evaluated using the MTT test [31,32] on HaCaT cells grown in Dulbecco’s modified Eagle’s medium with high glucose (DMEM) (Lonza, Milano, Italy), supplemented with 10% FBS (fetal bovine serum), 100 U/mL penicillin, 100 μg/mL streptomycin, and 2 mM l-glutamine. Cells were incubated at 37 °C for 24 h in 95% air/5% CO_2_ until 80% confluence. Liposome formulations were dispersed in cell culture medium and diluted 1:50, 1:100, 1:200, and 1:500, corresponding to liposome concentrations of 0.5, 0.25, 0.125, and 0.05 mg/mL, respectively. Seeded cells were exposed to the selected formulations for 24 h, and, after complete removal of the treatment, 110 μL of MTT (0.5 mg/mL) was added and incubated for 4 h. The conversion of MTT solution into a violet-colored formazane was obtained after addition, incubation (15 min), and shaking of 100 μL of DMSO. The solution absorbance, proportional to the number of living cells, was measured using a spectrophotometer at 590 nm and converted into percentage viability.

Statistical analysis was performed using analysis of variance (ANOVA). The level of significance was taken at *p* < 0.05.

## 4. Conclusions

In this study, the possible use of manganese derivatives for diagnostic applications in multimodal imaging techniques such as positron emission tomography (PET) and magnetic resonance imaging (MRI) was investigated. As manganese(II/III) complexes, MP, MDE2, MDB, and MDE3 were considered due to their different water solubility. In particular, two different types of anionic liposomes carrying the different manganese complexes were prepared and analyzed in terms of morphology, size, and magnetic power, together with the loading capacity and in vitro activity. This study enabled the selection of liposomes based on SLL as the best in terms of size, encapsulation, retention, and physical stability over time. Moreover, LS did not show differences in loading hydrophilic or lipophilic manganese compounds, resulting in a suitable system to convey all the synthesized complexes. In addition, LS displayed good results in terms of safety toward cells and ability to cross the cell membrane. The encouraging results obtained concerning the magnetic properties after LS encapsulation allowed to select MBD as the manganese compound to be further investigated for a potential application in diagnostic imaging. Remarkably, the possibility to administer the manganese complex using liposomes can represent an innovative approach to hamper the toxicity issues related to manganese administration. The preliminary data shown in this study strongly support further preclinical studies aimed at understanding the potential applicability of these formulations for diagnostic purposes.

## Figures and Tables

**Table 1:** Magnetic data of the indicated Mn complexes.

Compound	Mass Susceptibility χ (emu/g Oe) × 10^−5^ at T = 300 K	θ (K)	Moment μ_eff_ (BM)
**MnCl_2_** (reference)	7.2 ± 0.2	−7.0 ± 0.3	5.9 ± 0.3
**MP**	3.2 ± 0.1	−5.0 ± 0.3	6.1 ± 0.3
**MDE2** (fresh)	3.7 ± 0.1	−156 ± 8	6.2 ± 0.3
**MDE2** (aged)	1.51 ± 0.05	−23 ± 1	3.4 ± 0.2
**MDB** (fresh)	2.80 ± 0.08	−34 ± 2	6.5 ± 0.3
**MDB** (aged)	1.76 ± 0.05	−13 ± 1	5.0 ± 0.3

**Table 2:** Composition of the liposomal formulations considered in the present study.

Liposome Dispersions Acronym	PC (mg/mL)	CH (mg/mL)	Anionic Surfactant (mg/mL)	MP (µM)		MDE2 (µM)		MDE3 (µM)		MDB (µM)	
				t ^a^	ex ^b^	t ^a^	ex ^b^	t ^a^	ex ^b^	t ^a^	ex ^b^
**LN**	18.5	4.60	1.75	-	-	-	-	-	-	-	-
**LS**	18.5	4.50	2.00	-	-	-	-	-	-	-	-
**LN-MP**	18.5	4.60	1.75	500	427	-	-	-	-	-	-
**LS-MP**	18.5	4.50	2.00	500	471	-	-	-	-	-	-
**LN-MDE2**	18.5	4.60	1.75	-	-	500	169	-	-	-	-
**LS-MDE2**	18.5	4.50	2.00	-	-	500	413	-	-	-	-
**LN-MDE3**	18.5	4.60	1.75	-	-	-	-	500	241	-	-
**LS-MDE3**	18.5	4.50	2.00	-	-	-	-	500	426	-	-
**LN-MDB**	18.5	4.60	1.75	-	-	-	-	-	-	500	317
**LS-MDB**	18.5	4.50	2.00	-	-	-	-	-	-	500	446

^a^ Theoretical Mn concentration; ^b^ experimental Mn concentration.

**Table 3:** Dimensional parameters of extruded liposomes containing manganese complexes in time.

Time *Days*	LN-MP	LS-MP	LN-MDE2	LS-MDE2	LN-MDE3	LS-MDE3	LN-MDB	LS-MDB
	Mean Size (nm ± SD)	Mean Size (nm ± SD)	Mean Size (nm ± SD)	Mean Size (nm ± SD)	Mean Size (nm ± SD)	Mean Size (nm ± SD)	Mean Size (nm ± SD)	Mean Size (nm ± SD)
	*PDI ± SD*	*PDI ± SD*	*PDI ± SD*	*PDI ± SD*	*PDI ± SD*	*PDI ± SD*	*PDI ± SD*	*PDI ± SD*
0	188.5 ± 2.1	192.3 ± 1.9	287.4 ± 1.3	154.5 ± 4.2	187.6 ± 1.9	185.5 ± 3.5	185.7 ± 6.5	186.7 ± 7.3
	*0.09 ± 0.00*	*0.10 ± 0.03*	*0.36 ± 0.10*	*0.09 ± 0.01*	*0.111 ± 0.010*	*0.11 ± 0.03*	*0.10 ± 0.01*	*0.11 ± 0.01*
1	192.5 ± 3.2	192.4 ± 1.8	294.6 ± 6.3	154.1 ± 1.2	188.4 ± 1.3	185.9 ± 2.3	187.9 ± 5.2	188.7 ± 9.1
	*0.01 ± 0.03*	*0.09 ± 0.01*	*0.36 ± 0.033*	*0.09 ± 0.01*	*0.114 ± 0.014*	*0.08 ± 0.01*	*0.09 ± 0.01*	*0.09 ± 0.01*
3	188.8 ± 4.8	192.2 ± 1.6	278.8 ± 3.4	154.3 ± 5.3	189.3 ± 2.3	187.5 ± 3.4	186.9 ± 4.4	190.4 ± 6.3
	*0.13 ± 0.02*	*0.10 ± 0.01*	*0.35 ± 0.03*	*0.12 ± 0.04*	*0.086 ± 0.011*	*0.08 ± 0.03*	*0.11 ± 0.02*	*0.08 ± 0.01*
7	190.7 ± 2.3	190.7 ± 1.4	284.4 ± 2.3	154.7 ± 2.1	188.4 ± 1.3	187.5 ± 2.1	185.8 ± 4.9	191.1 ± 2.4
	*0.10 ± 0.03*	*0.11 ± 0.04*	*0.34 ± 0.04*	*0.09 ± 0.03*	*0.077 ± 0.028*	*0.10 ± 0.03*	*0.10 ± 0.01*	*0.09 ± 0.01*
15	193.5 ± 4.3	194.7 ± 2.3	292.9 ± 2.8	157.6 ± 1.1	187.3 ± 0.9	182.6 ± 0.9	186.3 ± 3.89	189.1 ± 4.5
	*0.12 ± 0.01*	*0.08 ± 0.04*	*0.37 ± 0.03*	*0.12 ± 0.02*	*0.04 ± 0.01*	*0.11 ± 0.03*	*0.10 ± 0.01*	*0.10 ± 0.01*
30	190.9 ± 1.3	192.4 ± 1.9	271.2 ± 1.09	155.8 ± 1.6	186.1 ± 1.5	181.9 ± 1.9	184.0 ± 2.2	188.5 ± 3.1
	*0.10 ± 0.01*	*0.10 ± 0.02*	*0.29 ± 0.02*	*0.10 ± 0.00*	*0.09 ± 0.06*	*0.12 ± 0.02*	*0.09 ± 0.01*	*0.09 ± 0.01*
60	192.5 ± 1.7	191.3 ± 4.3	438.6 ± 5.3	151.7 ± 1.8	184.3 ± 2.0	183.5 ± 1.6	194.4 ± 6.2	196.9 ± 3.3
	*0.10 ± 0.01*	*0.11 ± 0.01*	*0.35 ± 0.12*	*0.15 ± 0.04*	*0.10 ± 0.02*	*0.11 ± 0.03*	*0.12 ± 0.01*	*0.12 ± 0.03*

## Data Availability

Not applicable.

## Supplementary Materials

The following are available online at https://www.mdpi.com/article/10.3390/molecules27103124/s1: Figure S1: Curve of mass susceptibility (χ) as a function of temperature (T) measured on a sample of MnCl_2_, in H_appl_ = 10 kOe. The red line is the fitting curve to the Curie–Weiss law.

## Sample Availability

Samples of the compounds are available from the authors.
