# Supplementary material for: Synthetic and Nanotechnological Approaches for a Diagnostic Use of Manganese
Source: Molecules. 2022 May 13;27(10):3124. doi: 10.3390/molecules27103124 (PMC9146667; doi:10.3390/molecules27103124)
Supplement: Supplementary file 1 [file molecules-27-03124-s001.zip › molecules-1716657-supplementary.pdf]

molecules

MDPI

Supplementary Materials

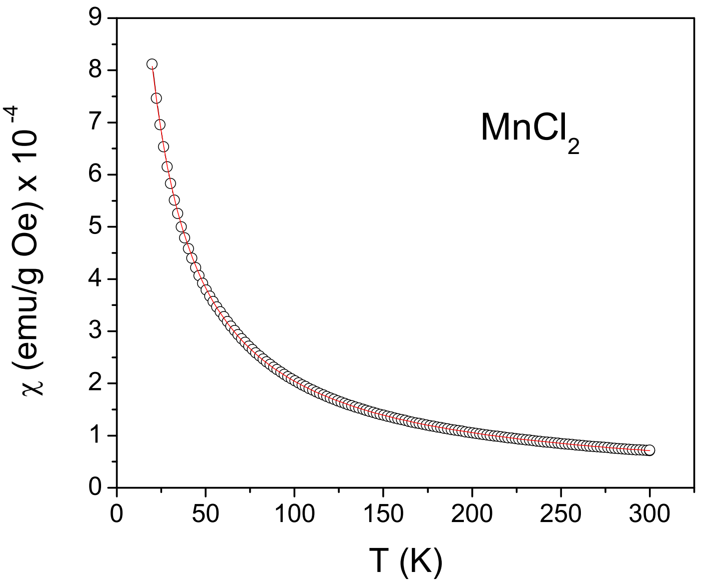

Figure S1. Curve of mass susceptibility (χ) as a function of temperature (T) measured on a sample of $MnCl_2$, in H_{appl} = 10 kOe. The red line is the fitting curve to the Curie-Weiss law.
